# The persistence of cognitive biases in financial decisions across economic groups

**DOI:** 10.1038/s41598-023-36339-2

**Published:** 2023-06-26

**Authors:** Kai Ruggeri, Sarah Ashcroft-Jones, Giampaolo Abate Romero Landini, Narjes Al-Zahli, Natalia Alexander, Mathias Houe Andersen, Katherine Bibilouri, Katharina Busch, Valentina Cafarelli, Jennifer Chen, Barbora Doubravová, Tatianna Dugué, Aleena Asfa Durrani, Nicholas Dutra, Eduardo Garcia-Garzon, Christian Gomes, Aleksandra Gracheva, Neža Grilc, Deniz Mısra Gürol, Zoe Heidenry, Clara Hu, Rachel Krasner, Romy Levin, Justine Li, Ashleigh Marie Elizabeth Messenger, Melika Miralem, Fredrik Nilsson, Julia Marie Oberschulte, Takashi Obi, Anastasia Pan, Sun Young Park, Daria Stefania Pascu, Sofia Pelica, Maksymilian Pyrkowski, Katherinne Rabanal, Pika Ranc, Žiga Mekiš Recek, Alexandra Symeonidou, Olivia Symone Tutuska, Milica Vdovic, Qihang Yuan, Friederike Stock

**Affiliations:** 1 Department of Health Policy and Management, Columbia University, 722 W 168th Street, New York, NY 10032 USA; 2 Centre for Business Research, Judge Business School, University of Cambridge, Cambridge, CB2 1AG UK; 3 Department of Experimental Psychology, University of Oxford, Oxford, UK; 4 Department of Psychology, University of Padua, Via VIII Febbraio, 35122 Padua, PD Italy; 5 Department of Computer Science, Columbia University, 500 W 120th Street, New York, NY 10027 USA; 6 Department of Psychology, Columbia University, 1180 Amsterdam Avenue, New York, NY 10027 USA; 7 Columbia University, 116th and Broadway, New York, NY 10027 USA; 8 Department of Psychology and Behavioural Sciences, Aarhus University, Bartholins Allé 11, 8000 Aarhus, Denmark; 9 German Center for Addiction Research in Childhood and Adolescence, University Medical Center Hamburg-Eppendorf, Martinistr 52, 20246 Hamburg, Germany; 10 Department of Economics, Columbia University, 420 W 118th Street, New York, NY 10027 USA; 11 Department of Psychology, Faculty of Social Studies, Masaryk University, Joštova 218/10, 60200 Brno, Czech Republic; 12 Department of Health, Universidad Camilo José Cela, Madrid, Spain; 13 Department of Political Science, Columbia University, 420 W 118th Street, New York, NY 10027 USA; 14 Department of Life Sciences, University of Roehampton, Whitelands College, London, SW15 4JD UK; 15 Department of Psychology, Koc University, 34349 Istanbul, Turkey; 16 Department of Psychology, Barnard College, Columbia University, 3009 Broadway, New York, NY 10027 USA; 17 Department of Biological Sciences, Columbia University, 1212 Amsterdam Avenue, New York, NY 10027 USA; 18 Department of Psychology, University of Stirling, Stirling, Scotland FK9 4L UK; 19 Lund University, Lund, Sweden; 20 Department of Clinical Neuroscience, Division of Psychology, Karolinska Institutet, Solna, 171 77 Stockholm, Sweden; 21 Department of Psychology, Ludwig-Maximilians-Universität München, Munich, Germany; 22 Department of Public Administration, Columbia University, 420 West 118th Street, New York, NY 10027 USA; 23 Department of Developmental Psychology and Socialisation, University of Padua, Via Venezia 12, 35131 Padua, PD Italy; 24 Department of Social and Organizational Psychology, Iscte-University Institute of Lisbon, Avenida das Forças Armadas, 1649-026 Lisbon, Portugal; 25 SWPS University, Chodakowska 19/31, Warsaw, Poland; 26 Department of Cognitive Science, Columbia University, 116th & Broadway, New York, NY 10027 USA; 27 Department of Psychology, University of Ljubljana, Aškerčeva Cesta 2, 1000 Ljubljana, Slovenia; 28 Department of Clinical Psychology, Leiden University, Wassenaarseweg 52, 2333 AK Leiden, South Holland The Netherlands; 29 Department of Sociology, Columbia University, 606 W 122nd Street, New York, NY 10027 USA; 30 Department of Psychology, Faculty of Media and Communications, Singidunum University, Karadjordjeva 65, Belgrade, 11000 Serbia; 31 University of Cologne, Albertus-Magnus-Platz, 50923 Cologne, Germany

**Keywords:** Psychology, Human behaviour

## Abstract

While economic inequality continues to rise within countries, efforts to address it have been largely ineffective, particularly those involving behavioral approaches. It is often implied but not tested that choice patterns among low-income individuals may be a factor impeding behavioral interventions aimed at improving upward economic mobility. To test this, we assessed rates of ten cognitive biases across nearly 5000 participants from 27 countries. Our analyses were primarily focused on 1458 individuals that were either low-income adults or individuals who grew up in disadvantaged households but had above-average financial well-being as adults, known as positive deviants. Using discrete and complex models, we find evidence of no differences within or between groups or countries. We therefore conclude that choices impeded by cognitive biases alone cannot explain why some individuals do not experience upward economic mobility. Policies must combine both behavioral and structural interventions to improve financial well-being across populations.

## Introduction

Economic inequality is a direct and global barrier to upward mobility and positive socioeconomic outcomes, perpetuating negative effects for individual and population health, well-being, and sustainability^1^. In broad terms, economic mobility is a measurement of substantive change in financial well-being status, such as going from middle-class to wealthy or poverty to low-income. In this paper, economic mobility is understood beyond simply income, but through comparative financial security, such as wealth, debt, employment opportunity, and ability to withstand economic shocks.

Within countries, economic inequality continues to rise, made worse by the COVID-19 pandemic, disrupting decades of improvement in which inequality between countries had declined^2^. There are myriad established links between economic inequality and decision-making, such as how individuals from disadvantaged communities are more prone to higher rates of impulsivity^3^ and temporal discounting^4^. Individual factors such as existing wealth and education are known to influence financial decision-making^5^. However, while it is widely studied that such financial behaviors may be influenced by cognitive biases (e.g., familiarity heuristics^6,7^, optimism^8^, proximity^8^), there is an absence of definitive evidence whether individual decision-making ability is directly associated with upward economic mobility on a population level. There is a general view that poverty leads to attentional focus on scarcity demands, which amplifies biases such as risk aversion^9^.

However, others^10–12^ argue that low-income individuals are not substantively different in decision-making^13^, but instead face narrower margins^14^ and greater impact from their immediate environment. There is also evidence to suggest that even making generally good financial decisions can have differentiated outcomes for low-income individuals due to large transactional costs against relatively small investment gains^15^. Inequality is also associated with lower self-belief in achieving socio-economic success, diminishing the motivation to engage in behaviors associated with long-term socioeconomic growth^1^.

Behavioral interventions have attempted to reduce inequalities by informing individuals of the decisions associated with better socioeconomic outcomes. For instance, the Swedish government’s opt-out pension plan for workers facilitated better pension investment strategies^16^. Similarly, across several studies from different countries, Reñosa et al.^17^ found that vaccination hesitancy was lower following simple behavioral nudges that made information more salient or were linked to incentives. In Kenya, unconditional cash transfers (UCTs) promoted better socioeconomic outcomes by enabling the coverage of immediate costs and the investment of any excess funds (e.g. in durable assets or business activities)^18^.

Unfortunately, despite some positive effects, behavioral approaches to reducing economic inequality have been largely ineffective at making substantive impacts. This may be due to measures focused on the modal person without considering marginalized groups. Consider three examples: first, the U.S. Earned Income Tax Credit aims to help low- to moderate-income workers reduce their tax burden, yet is under-subscribed by those that stand to benefit the most. When state agencies and non-profit organizations attempted established behavioral nudges to promote the utilization of and access to credits among the lowest-income families, effects were null and even linked to distrust among targeted groups^19^. Similarly, a large UCT experimental trial in the U.S. was followed by worsened subjective financial and psychological outcomes, rather than indicating positive benefits amongst recipients^20^. A conditional cash transfer program in Indonesia failed to support the needs of the lowest-income beneficiaries due to inadequate distribution of funds^21^. The incongruent effects between economic classes of such programs are a strong indication of need for new approaches.

“Positive deviance” is a framework which studies individuals from disadvantaged circumstances that experience notably better outcomes or routinely make more optimal choices than similarly disadvantaged peers^22^. Positive deviance approaches focus on understanding observed behaviors of individuals, thus lending practical policy suggestions^23^. As general interventions for improving financial well-being may inadvertently backfire among underprivileged groups^24^, considering the patterns of positive deviants may aid in developing programs with more successful impact.

While positive deviance has been identified around the world^25–28^ no substantive work across countries and economic contexts exists to determine its viability as a frame for research or policy design. To ensure reliability, replicability, and generalizability prior to proposing a new construct for explaining behavior, there is considerable value in taking a multi-country, large-sample approach^29^. This both limits methodological biases based on sample or language^30^ and presents more globalized contours of psychological and behavioral constructs^12^.

**Figure 1:**
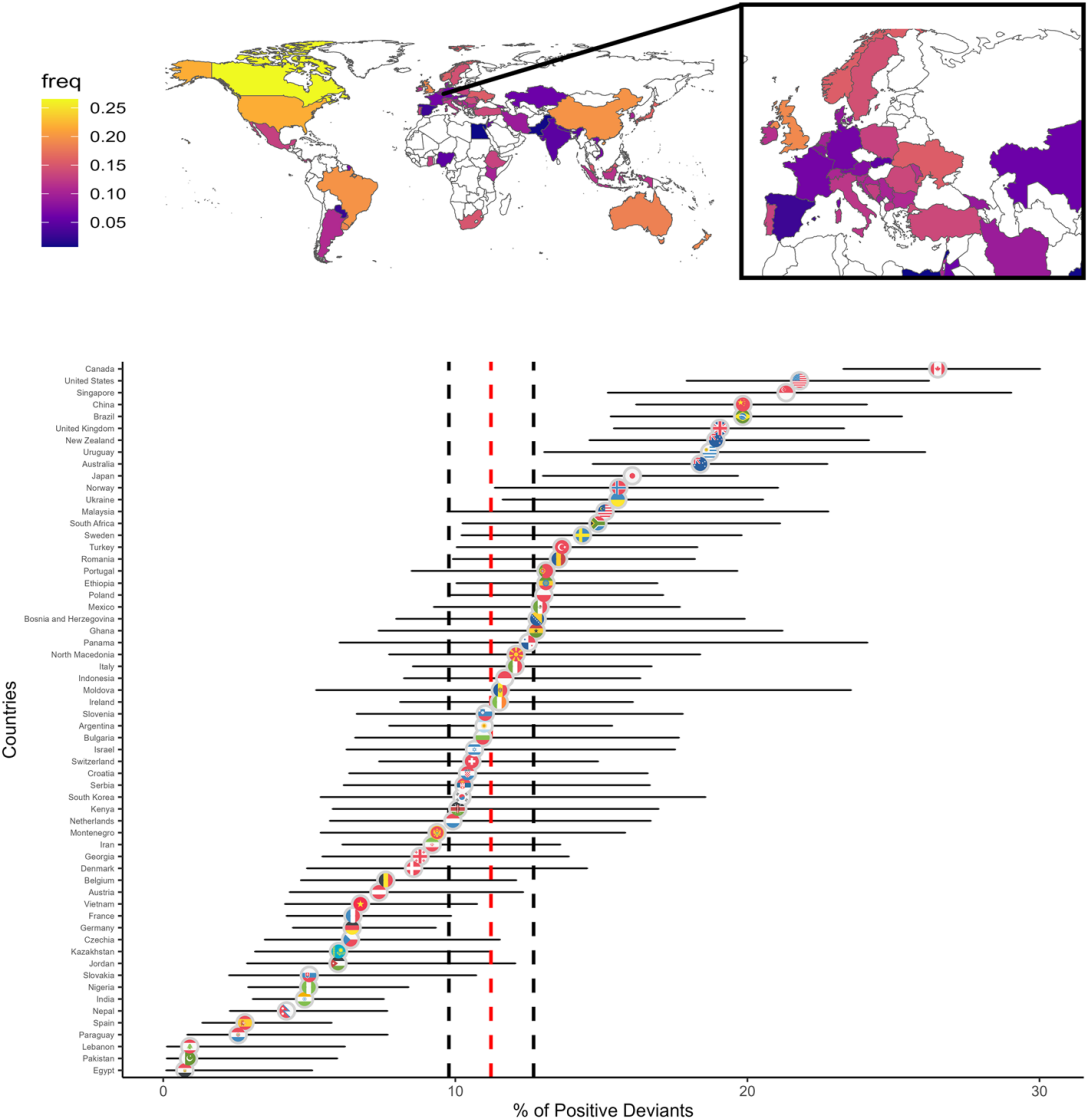
Frequency of positive deviance across country samples with a cross section on Europe, as taken from Ruggeri et al.^12^. Map generated with rnaturalearth.

Secondary analysis of data from 60 countries^12^ shows that rates of positive deviance are highly varied (Fig. 1), indicating a number of potential environmental and/or individual factors may contribute to population-level mobility. While most work on such economic matters will understandably focus on incomes, employment, education, and other systemic factors, how individuals make decisions under scarcity will also help develop more effective policies (in response to those failed attempts described).

One low-cost way to test potential differences in decision-making on a large scale is through cognitive biases known to influence (and harm) decision-making, particularly financial choices^31^. Assessing cognitive biases across multiple countries helps assess if patterns of preferences reflect specific environments and if choice patterns are highly similar but barriers impede consistent outcomes. If biases emerge consistently and vary between positive deviants and those who remain low-income, upward economic movement may be heavily explained by choices. If no such patterns emerge, it strongly suggests that barriers and absence of resources or opportunities are the most critical indicators of outcomes rather than unique choice patterns. Focusing our assessment on a global sample provides a robust insight compared to presenting findings from a single location and making assumptions about large applicability. With this approach, meaningful results may address economic inequalities in different settings.

The aim of this study was to test whether cognitive biases were observed at different rates between positive deviants and those who remain in disadvantaged circumstances as adults. Because of varying economic systems, we attempted to engage participants from around the world to produce a more robust first attempt at this research approach, rather than only those in similar environments. This was important in terms of added value of this research because most biases used here have primarily been tested and validated in contexts considered to meet the WEIRD (Western, Educated, Industrialized, Rich, Democratic) classification.

We expected to identify small to moderate differences in cognitive biases between positive deviants and low-income adults, looking at both the full sample as well as analyzing within each country. We also anticipated heterogeneity in differences in proportions of cognitive biases between countries. As this was the first such approach on the topic, some aspects were highly exploratory and we planned to report general patterns even if in the opposite direction than anticipated (i.e., if there were certain biases more common among positive deviants).

Ultimately, the primary research question was to understand if some individuals may overcome extremely disadvantaged financial circumstances in part due to resistance against cognitive biases that may impede optimal decision-making. If so, it may explain why some behavioral interventions aimed at reducing inequality have been unsuccessful. However, if no substantive differences exist, it would give strong evidence against the idea that individuals remain poor through choices alone. It would also indicate a more robust understanding of human behavior is necessary to develop effective policies for meaningful impact across populations.

## Results

To test our pre-registered hypotheses (osf.io/wj9yn), we ran binomial logistic regressions to predict differences in the presence of individual cognitive biases between positive deviants and low-income individuals (we mostly ignore comparisons with high-income individuals for this research, though data are available for such use). Bayesian meta-analyses were used to assess overall presentation of cognitive biases to account for potential heterogeneity within countries. Pooled Bayesian meta-analysis checked for differences among positive deviants across countries.

**Figure 2:**
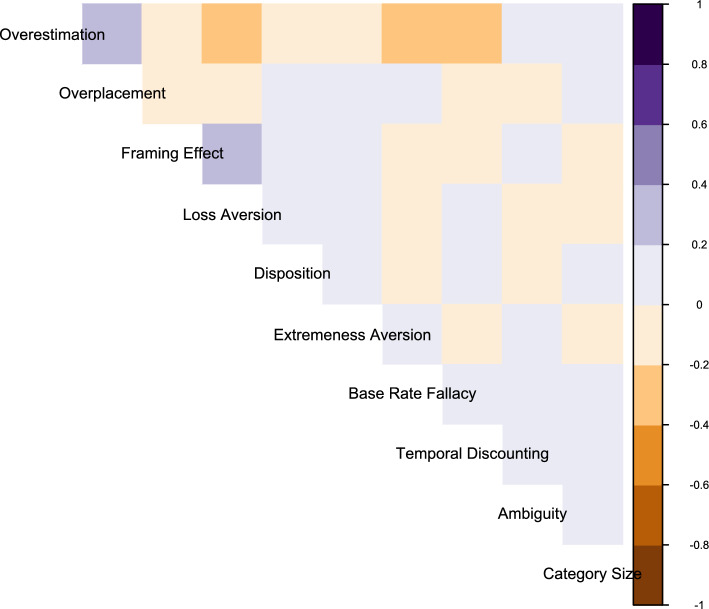
Correlation between ten biases within 3346 participants showed each bias was largely unique and not collinear with other biases assessed, with the exception of overplacement and overestimation (which rely on the presence of some biases).

Across ten cognitive biases, rates observed ranged from 28.2% (temporal discounting) to 70% (ambiguity bias). On average, participants exhibited 3.23 (*SD* = 1) cognitive biases. As indicated in Fig. 2, individual biases were not highly correlated within individuals, which is why we treated them in discrete analysis rather than creating an index.

### Rates of cognitive biases between income groups

**Figure 3:**
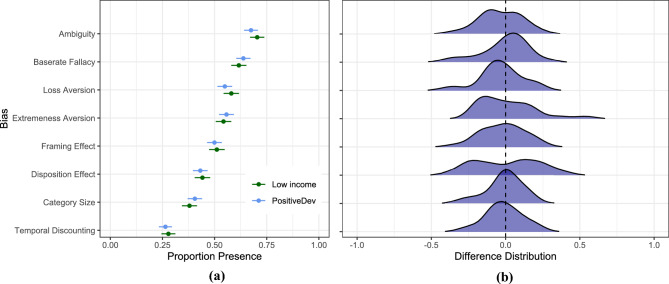
(**A**) Presence of cognitive biases for low income and positive deviant participants for the pooled sample and (**B**) distribution of country mean differences in observed biases between low income participants and positive deviants. Note that overplacement and overestimation are not included as they are measured in counts rather than proportion.

Chi-squared tests showed no significant differences between the rates of any of the eight cognitive biases demonstrated by low-income individuals or positive deviants, as indicated in Fig. 3A,B (see also Supplementary Material, Table S8). Next, we conducted binomial logistic regressions to predict the presence of cognitive bias based on income group and country of residence. Prediction coefficients were not significant in any of ten logistic regressions; positive deviants were equally likely to exhibit cognitive bias compared to low-income individuals (see Table 1). As a robustness check, we also ran complementary Bayesian logistic regressions, whose results are consistent with these. We conclude that this additional analysis provides further evidence that rates of cognitive biases do not seem to differ between positive deviants and low-income adults. A table reporting credible intervals from all Bayesian logistic regressions can be found in the Supplementary Materials (Table S6).

**Table 1:** Logistic regression coefficients for predicting biases by residence and income group.

	N	Disposition	Ambiguity	Base rate	Category size	Extremeness	Temporal discount	Framing	Loss aversion	N	Overplacement	Overestimation
(Intercept)	132	− 0.466* [− 0.847, − 0.094]	0.719* [0.338, 1.112]	0.182 [− 0.187, 0.554]	− 0.592* [− 0.98, − 0.216]	0.085 [− 0.281, 0.452]	− 0.998*[− 1.431, − 0.589]	0.278 [− 0.089, 0.649]	0.595* [0.221, 0.98]	66	− 0.509 [− 1.037, 0.001]	− 0.068 [− 0.576, 0.438]
Positive deviance	NA	− 0.047 [− 0.265, 0.171]	− 0.152 [− 0.386, 0.081]	0.051 [− 0.176, 0.278]	0 [− 0.222, 0.221]	0.011 [− 0.206, 0.227]	− 0.169 [− 0.415, 0.077]	− 0.056 [− 0.271, 0.159]	− 0.112 [− 0.334, 0.109]	NA	0.139 [− 0.126, 0.405]	0.014 [− 0.25, 0.278]
Bosnia and Herzegovina	80	− 0.68* [− 1.321, − 0.067]	0.023 [− 0.561, 0.617]	0.962* [0.355, 1.6]	0.239 [− 0.335, 0.81]	− 1.188* [− 1.817, − 0.589]	0.452 [− 0.158, 1.06]	− 0.403 [− 0.966, 0.154]	0.019 [− 0.557, 0.602]	NA	NA	NA
Brazil	75	0.025 [− 0.564, 0.607]	0.82* [0.156, 1.531]	0.741* [0.14, 1.366]	0.351 [− 0.23, 0.932]	0.098 [− 0.471, 0.671]	0.719* [0.112, 1.33]	− 0.441 [− 1.016, 0.129]	0.087 [− 0.503, 0.688]	75	0.912* [0.239, 1.602]	− 0.455 [− 1.134, 0.216]
Canada	66	0.432 [− 0.166, 1.032]	− 0.073 [− 0.686, 0.551]	− 0.271 [− 0.867, 0.321]	0.592 [− 0.007, 1.196]	0.672* [0.06, 1.305]	− 0.315 [− 1.066, 0.391]	0.122 [− 0.475, 0.727]	− 0.715* [− 1.321, − 0.118]	NA	NA	NA
Chile	22	− 1.356* [− 2.84, − 0.217]	1.205 [0.064, 2.691]	1.297* [0.248, 2.579]	0.033 [− 0.947, 0.954]	− 0.272 [− 1.198, 0.635]	0.319 [− 0.715, 1.273]	− 0.809 [− 1.786, 0.106]	− 0.722 [− 1.653, 0.189]	NA	NA	NA
Czech Republic	73	0.073 [− 0.518, 0.658]	− 0.108 [− 0.702, 0.493]	1.322* [0.655, 2.046]	0.345 [− 0.241, 0.93]	0.502 [− 0.081, 1.099]	0.107 [− 0.552, 0.751]	− 0.609* [− 1.195, − 0.033]	0.507 [− 0.114, 1.154]	73	0.021 [− 0.661, 0.706]	− 0.983* [− 1.709, − 0.28]
Denmark	51	0.529 [− 0.124, 1.185]	− 0.287 [− 0.949, 0.384]	− 0.01 [− 0.66, 0.645]	0.071 [− 0.611, 0.736]	− 0.208 [− 0.86, 0.44]	− 0.462 [− 1.333, 0.327]	− 0.211 [− 0.861, 0.44]	− 0.102 [− 0.762, 0.572]	51	0.244 [− 0.498, 0.989]	− 0.135 [− 0.873, 0.598]
Germany	44	0.396 [− 0.297, 1.087]	0.329 [− 0.407, 1.114]	− 0.022 [− 0.708, 0.672]	0.225 [− 0.485, 0.921]	0.47 [− 0.224, 1.191]	− 1.235* [− 2.492, − 0.232]	− 1.123* [− 1.885, − 0.407]	− 0.456 [− 1.149, 0.238]	NA	NA	NA
Greece	51	0.607 [− 0.045, 1.266]	0.648 [− 0.084, 1.444]	1.475* [0.691, 2.369]	− 0.283 [− 1.004, 0.404]	− 0.286 [− 0.942, 0.361]	0.723* [0.035, 1.408]	− 0.053 [− 0.703, 0.602]	− 3.741* [− 5.579, − 2.51]	51	1.135* [0.383, 1.917]	0.755 [0.007, 1.526]
Ireland	34	1.516* [0.708, 2.402]	− 0.022 [− 0.8, 0.792]	− 0.692 [− 1.485, 0.07]	− 0.757 [− 1.732, 0.101]	0.266 [− 0.492, 1.046]	0.22 [− 0.651, 1.038]	− 0.481 [− 1.253, 0.275]	− 0.886* [− 1.673, − 0.124]	34	− 0.595 [− 1.542, 0.29]	0.06 [− 0.771, 0.892]
Italy	45	1.185* [0.486, 1.919]	0.264 [− 0.457, 1.028]	0.386 [− 0.305, 1.103]	0.093 [− 0.62, 0.788]	− 0.313 [− 1.001, 0.365]	− 0.3 [− 1.179, 0.499]	− 0.561 [− 1.256, 0.118]	− 0.849* [− 1.55, − 0.165]	NA	NA	NA
Japan	88	0.295 [− 0.26, 0.85]	− 0.124 [− 0.693, 0.45]	− 0.753* [− 1.32, − 0.198]	− 0.506 [− 1.124, 0.091]	0.004 [− 0.543, 0.552]	− 1.02* [− 1.838, − 0.277]	0.295 [− 0.262, 0.86]	0.355 [− 0.228, 0.953]	88	− 0.504 [− 1.196, 0.182]	0.34 [− 0.306, 0.991]
North Macedonia	41	− 0.063 [− 0.807, 0.655]	0.232 [− 0.514, 1.024]	0.927* [0.164, 1.762]	− 0.064 [− 0.823, 0.663]	0.256 [− 0.45, 0.978]	0.625 [− 0.128, 1.364]	− 0.4 [− 1.112, 0.304]	− 0.595 [− 1.309, 0.114]	41	0.301 [− 0.49, 1.094]	− 0.084 [− 0.871, 0.698]
Oman	46	0.228 [− 0.46, 0.908]	0.776 [− 0.001, 1.639]	1.072* [0.323, 1.897]	0.418 [− 0.268, 1.1]	− 0.003 [− 0.676, 0.673]	0.734* [0.02, 1.442]	− 0.248 [− 0.924, 0.426]	− 0.537 [− 1.219, 0.142]	46	− 0.391 [− 1.207, 0.4]	− 0.473 [− 1.254, 0.29]
Peru	32	− 0.148 [− 0.987, 0.647]	1.074* [0.127, 2.207]	1.249* [0.355, 2.29]	1.381* [0.576, 2.247]	− 0.092 [− 0.87, 0.686]	1.239* [0.442, 2.051]	− 0.619 [− 1.422, 0.159]	− 0.393 [− 1.175, 0.395]	32	0.665 [− 0.186, 1.536]	0.059 [− 0.79, 0.909]
Poland	33	0.312 [− 0.468, 1.083]	− 0.066 [− 0.85, 0.752]	− 0.031 [− 0.797, 0.746]	− 0.101 [− 0.937, 0.69]	0.469 [− 0.306, 1.281]	0.54 [− 0.293, 1.343]	− 0.549 [− 1.336, 0.217]	− 0.833* [− 1.625, − 0.063]	33	0.119 [− 0.74, 0.968]	− 0.245 [− 1.1, 0.594]
Portugal	95	0.3 [− 0.236, 0.838]	1.034* [0.395, 1.719]	0.615* [0.064, 1.179]	− 0.384 [− 0.968, 0.186]	0.587* [0.046, 1.14]	− 0.372 [− 1.036, 0.266]	− 0.313 [− 0.845, 0.217]	− 0.349 [− 0.888, 0.189]	95	0.629 [− 0.005, 1.276]	0.251 [− 0.378, 0.884]
Romania	26	− 0.146 [− 1.065, 0.717]	0.357 [− 0.544, 1.358]	0.103 [− 0.743, 0.974]	1.062* [0.209, 1.959]	− 0.09 [− 0.939, 0.759]	0.772 [− 0.119, 1.642]	0.386 [− 0.473, 1.303]	0.097 [− 0.767, 1.017]	26	0.44 [− 0.478, 1.363]	− 0.409 [− 1.359, 0.507]
Serbia	33	− 0.486 [− 1.374, 0.329]	− 0.687 [− 1.467, 0.086]	1.099* [0.243, 2.072]	0.033 [− 0.784, 0.815]	0.469 [− 0.306, 1.281]	0.669 [− 0.149, 1.466]	− 0.937* [− 1.769, − 0.154]	− 1.221* [− 2.058, − 0.434]	33	0.364 [− 0.482, 1.213]	0.491 [− 0.351, 1.357]
Slovenia	86	− 0.125 [− 0.697, 0.439]	− 0.037 [− 0.603, 0.536]	0.458 [− 0.102, 1.029]	0.732* [0.181, 1.291]	0.532 [− 0.023, 1.099]	0.109 [− 0.519, 0.726]	0.336 [− 0.22, 0.901]	0.213 [− 0.357, 0.793]	86	− 0.163 [− 0.827, 0.502]	− 1.135* [− 1.845, − 0.448]
South Korea	42	0.114 [− 0.607, 0.82]	1.393* [0.471, 2.512]	0.267 [− 0.437, 0.994]	1.395* [0.668, 2.167]	0.393 [− 0.309, 1.12]	0.315 [− 0.471, 1.068]	0.456 [− 0.259, 1.206]	− 0.029 [− 0.739, 0.701]	42	− 0.284 [− 1.11, 0.52]	− 0.529 [− 1.34, 0.258]
Sweden	81	0.716* [0.157, 1.283]	− 0.409 [− 0.977, 0.158]	0.32 [− 0.243, 0.893]	0.269 [− 0.3, 0.836]	0.233 [− 0.323, 0.795]	− 0.563 [− 1.305, 0.13]	− 0.519 [− 1.082, 0.036]	0.107 [− 0.468, 0.692]	NA	NA	NA
Taiwan	39	0.019 [− 0.732, 0.747]	0.421 [− 0.357, 1.265]	0.051 [− 0.667, 0.782]	− 0.101 [− 0.879, 0.642]	0.273 [− 0.446, 1.011]	− 1.092 [− 2.353, − 0.082]	− 0.508 [− 1.24, 0.209]	− 1.011* [− 1.765, − 0.285]	39	− 1.729* [− 3.021, − 0.674]	0.319 [− 0.474, 1.125]
Turkey	36	0.947* [0.2, 1.723]	0.629 [− 0.198, 1.55]	− 0.213 [− 0.956, 0.529]	0.593 [− 0.154, 1.342]	− 0.662 [− 1.445, 0.087]	1.673* [0.901, 2.483]	− 1.065* [− 1.886, − 0.298]	− 0.527 [− 1.275, 0.219]	NA	NA	NA
United Kingdom	107	0.51 [− 0.006, 1.03]	− 0.317 [− 0.844, 0.209]	− 0.116 [− 0.629, 0.397]	− 0.494 [− 1.066, 0.064]	0.23 [− 0.283, 0.747]	− 0.513 [− 1.171, 0.12]	0.308 [− 0.213, 0.835]	0.061 [− 0.469, 0.595]	107	0.037 [− 0.589, 0.67]	− 0.577 [− 1.207, 0.048]
Total	1458	–	–	–	–	–	–	–	–	1018	–	–

Regarding income group, participants in this analysis are either low-income or positive deviants. The ‘Positive Deviance’ variable in the table captures the behavior of positive deviants, with low-income as the baseline (high-income participants are not included in this analysis). Regarding residence, all country variables reflect participants’ country of residence, with the USA as the baseline (for disposition to loss aversion; Canada is the baseline for Overplacement and Overestimation since the USA is excluded from those analyses along with Bosnia and Herzegovina, Chile, Germany, Italy, Sweden, and Turkey).

To examine the optimal choice patterns between low-income and positive deviants, we calculated the mean difference between the overplacement score and number of presented biases. One-way ANOVA showed no significant difference; (*F*(2) = 0.281, *P* = 0.755). We conducted additional ANOVAs for each country and found no significant differences between the three groups.

Because there were no substantive differences between groups, there are no additional insights to report on our second hypothesis anticipating positive deviants would show more optimal choice patterns (see Supplementary Material).

### Rates of cognitive biases between countries

Our third hypothesis expected differences in biases between countries in a way that might highlight how specific systems interacted with choice patterns. For example, recent work^12^ indicated that temporal discounting is much higher in countries where inflation is extreme. In our case, we wanted to present limits and heterogeneity in differences that would be explained by local contexts, but we did not anticipate systematically different results given that not all biases should be context-dependent (e.g., category size bias). Context-dependent patterns, such as how temporal discounting rates in our data were substantially higher in Turkey, will be reported in a separate paper.

First, we found that within countries, positive deviants did not significantly differ in the probability of showing cognitive bias from either low-income, or high-income groups (see Supplementary Materials, Table S5). Next, we built four models (two with all countries for eight biases; two with only the countries where all 10 biases were assessed—see *Selection of Countries* for why seven countries were not included for overplacement and overestimation) using Bayesian meta-analysis to assess differences in probability of showing cognitive biases within countries, twice for the entire population (N = 3194) and twice with positive deviants only (N = 528). In all models, we found no significant differences in the probability of showing biases between countries (0.22 < τ < 0.43; 3.98 < SMD < 4.95; see Supplementary Materials, Table S4).

## Discussion

This study aimed to determine if rates of cognitive biases were different between positive deviants and low-income adults in a way that might explain some elements of what impedes or facilitates upward economic mobility. We anticipated finding small-to-moderate effects between groups indicating positive deviants were less prone to biases involving risk and uncertainty in financial choices. However, across a sample of nearly 5000 participants from 27 countries, of which 1458 were low-income or positive deviants, we find no evidence of any difference in the rates of cognitive biases—minor or otherwise—and no systematic variability to indicate patterns vary globally.

In sum, we find clear evidence that resistance to cognitive biases is not a factor contributing to or impeding upward economic mobility in our sample. Taken along with related work showing that temporal choice anomalies are tied more to economic environment rather than individual financial circumstances^12^, our findings are (unintentionally) a major validation of arguments (especially that of Bertrand, Mullainathan, and Shafir^11^) stating that poorer individuals are not uniquely prone to cognitive biases that alone explain protracted poverty. It also supports arguments that scarcity^14^ is a greater driver of decisions, as individuals of different income groups are equally influenced by biases and context-driven cues^13,32^.

What makes these findings particularly reliable is that multiple possible approaches to analyses had to be considered while working with the data, some of which were considered into extreme detail before selecting the optimal approach. As our measures were effective at eliciting biases on a scale to be expected based on existing research, and as there were relatively low correlations between individual biases (e.g., observing loss aversion in one participant is not necessarily a strong predictor of also observing any other specific bias), we conclude that there is no evidence from our sample to support that biases are directly associated with potentially harming optimal choices uniquely amongst low-income individuals.

Of course, though our sample was reasonably well powered, it is possible that our focus on two subsets of the overall population may have been too small to detect small effects. First, some perspective on this may be useful: ensuring that 17% of our sample met the criteria for being positive deviants indicated that our recruitment strategy was effective at finding a sufficient number of participants for a small (by rule) group within a population. When using existing datasets, this can yield as low as 1%^22^. However, since we did not want to over-represent a group, but instead have a reasonable reflection of groups while also sufficient samples for analyses, we were satisfied that overall we had over 750 participants meeting the criteria out of a total sample of nearly 5000. With that said, future work may wish to focus on expanding the sample of low income or positive deviant groups in case large samples yield small but significant effects. Given the consistency of our null findings, however, we do not speculate a likelihood for this.

We do not argue that behavior has no link to individuals overcoming or remaining in negative financial circumstances. On the contrary, it is very evident that biases do exist despite income levels, and that targeting those may be beneficial. However, we argue that further work is particularly necessary to understand why similar choice patterns do not lead to similar outcomes. If those patterns were validated and still produced differential outcomes, it would likely be a result of substantial system barriers and scarcity of opportunities^11,33^. If validated, it would provide even stronger arguments toward investment in substantive structural changes to reducing inequality, rather than assuming that individual changes can alone overcome broader barriers^34^. This again does not mean there is no place for individually targeted behavioral interventions, but that they should be developed in combination with those that involve addressing systems and barriers^35^.

### Limitations

This is one of the first large-scale studies on positive deviance tested between countries and using cognitive biases as a frame. Our approach is therefore limited by not having been previously validated and used items that only superficially elicit biases but not necessarily reflect behaviors in real-world settings. Also, frames used may not have been truly reflecting biases but simply a random preference set based on the options given. This was evident in the intended items on mental accounting, which were removed after the study began based on a later determination that the items did not measure the intended choice pattern as written. However, it may also be true of the category size bias measure, which showed essentially a 50-50 behavior and may not be especially useful.

We are also limited in how we identified income groups, both due to the self-report nature and that participants were typically higher income as adults. This may also be collinear with the number of positive deviants that identified as immigrants: if they were born in low-income communities but migrated to a high-income country, whether they should qualify as positive deviants may be up for further debate. Similarly, we only measure a narrow set of biases, which are each tested discretely, rather than in combination (or controlling for) other factors such as personality, resilience, numeracy, personal beliefs (e.g., political or religious), or financial literacy. Future work may find that factoring in these aspects may elucidate different patterns.

Given our findings, one advantage of this approach is that there does not appear to be a need for longitudinal study on if or when positive deviants shift decision-making styles. That approach is typically recommended in static studies, where it is unclear if choices would have been the same prior to achieving financial wealth. Our findings indicate this may not be mandatory. However, we have attempted to avoid speaking to absolutely generalizability from our findings. Though we have a large and diverse sample, as the first study of this type and a sample that was intentionally not representative in order to engage many low-income participants, we strongly encourage further, multi-site studies to validate (or refute) our findings.

## Conclusion

We sought to determine if individuals that had overcome low-income childhoods showed significantly different rates of cognitive biases from individuals that remained low-income as adults. We comprehensively reject our initial hypotheses and conclude that outcomes are not tied—at least not exclusively or potentially even meaningfully—to resistance to cognitive biases. Our research does not reject the notion that individual behavior and decision-making may directly relate to upward economic mobility. Instead, we narrowly conclude that biased decision-making does not alone explain a significant proportion of population-level economic inequality. Thus, any attempts to reduce economic inequality must involve both behavioral and structural aspects. Otherwise, similar decisions between disadvantaged individuals may not lead to similar outcomes. Where combined effectively, it will be possible to assess if genuine impact has been made on the financial well-being of individuals and populations.

## Methods

Ethical approval for this research was given by the Institutional Review Board at Columbia University. All methods were carried out following relevant guidelines and regulations. All country surveys were provided in at least one primary local language, as well as screened for cultural appropriateness, flow, and overall quality. Each participant provided informed consent to participate in the study. All materials and methods followed our pre-registered plan (osf.io/wj9yn), except for certain deviations, which are described later. Further details are provided in the Supplementary Materials.

### Selection of countries

There was no systematic approach to country inclusion, but we explicitly emphasized including some countries that are not typically represented in behavioral research. Countries were essentially chosen based on locations and languages where study volunteers were capable of recruiting substantive samples ethically (i.e., with reasonable oversight and appropriate methods). This means selection was not entirely at random, but there was no specific guiding criterion in which countries were included apart from representation in the study team. No country was added based on any unique factors, such as wealth, economic systems, or idiosyncratic contexts.

Following data collection, 27 countries were fully included, using 22 languages. Two countries were attempted but were unable to fulfill certain tasks or were removed for ethical concerns. Several countries (Bosnia and Herzegovina, Chile, Germany, Italy Sweden, Turkey, and the United States) were part of preliminary work in developing the full study. Participants in those countries answered slightly more questions, some of which were removed for the full study. For this reason, those countries are not included in the overestimation and overplacement analyses, as participants in those countries saw slightly different versions of the items.

### Translations

All survey instruments utilized forward-and-back translations for all countries in their primary language. At least one native speaker was involved throughout each process, requiring translation into local currencies (and cost standards) as well as applicable aspects such as race, education, and employment reporting standards. In some countries, varying demographic measures were modified for cultural and ethical appropriateness. Guidelines for race and ethnicity were observed in countries with specific rules, such as where racial identity questions are regulated or prohibited. Additional details and full surveys for each country can be found under the pre-registration link (osf.io/wj9yn).

### Instrument

To measure cognitive biases with implications for decision-making in financial situations, we used 15 decision items that assessed 10 cognitive biases. These items were selected following preliminary data from a parallel study that was pre-registered using the Open Science Framework (osf.io/hmk9s) prior to data collection. Following an exhaustive process in which a large number of biases were reviewed from multiple scientific repositories, biases used in this study were ultimately selected on several criteria. Biases had to be directly relevant to financial decision-making, sufficient at eliciting cognitive biases in a large sample using simple discrete choice methods, and not require long or complex statements. The final list of biases used was the ambiguity effect^36^, base rate fallacy^37^, category size bias^38^, extremeness aversion^39^, disposition effect^40^, temporal discounting^12^, overplacement bias^41^, overestimation bias^42^, framing effect^43^, and loss aversion^44^. The pre-registered study mentioned earlier details additional biases that were piloted separately from this study, but removed for lack of sufficiently meeting these criteria.

Biases and their associated items were also selected specifically to meet certain practical criteria related to ease of understanding and avoiding complications related to translations. For example, we did not use vignettes or lengthy statements on scenarios to present choices. Instead, we used the most direct and singular approaches that were possible. While this was not always perfectly doable, some potential measures were excluded if they were deemed to be overly complicated or if the specific aspects might have been unfamiliar to most participants. This was particularly true for items that would have presented complex financial options only known to financially active individuals. Finally, we did not select items that would implicitly or explicitly appear to relate to poverty on inequality. Instead, we chose items that would be relevant to any economic class, in a way that may elicit any differences in choice patterns between groups if such differences would explain differential economic outcomes.

Financial values were adapted to local currencies and income standards (see: osf.io/wj9yn for the information on financial values and supplementary information on them) The survey also includes employment, bill management, income, debit and credit circumstances, and socioeconomic status as a child. We also collect age, gender, education level, parent education level, race, and ethnicity (where permitted and appropriate).

### Procedure

Participant recruitment utilized Qualtrics surveying software to collect data. Most participants were recruited using the Demić-Većkalov method^12^, which included posting links on discussion threads and online news articles (social media, popular forums, and news websites). We also implemented the Jarke method of identifying popular communication media associated with specific groups that were not represented (e.g., rugby forums on social media to recruit males from New Zealand). The survey was also circulated to local non-governmental and non-profit organizations, and for-profit corporations to generate informal “snowballing.” Some participants were recruited by convenience sampling. Only residents of Japan were compensated (less than US$1 total). This approach helped to minimize sample bias across countries and generate diverse backgrounds among participants, with the main exception of mostly including populations with direct internet access and social media accounts.

Because this study requires internet access and largely relies on visibility on popular (but not universally used) platforms, the team made concerted efforts to make direct contact with organizations, institutions, and government agencies to recruit participants through different media. Some of these methods included contacting Human Resource officers at large employers in different countries and specifically requesting circulation among individuals from lower-income backgrounds. We also communicated with a number of NGOs and non-profits to see if they would recruit community members as participants if they visited their sites in order to use computers or access the internet.

After confirming eligibility and giving consent, participants were presented 15 binary choice scenarios. For example, to measure category size biases, participants were asked to choose if they would prefer a scenario with one winning ticket out of 10 or 10 winning tickets out of 100 (see Supplement Table S9). Decision-making items were shown in a randomized order, except for choices that required a specific sequence (such as overestimation being required to appear last). Financial and demographic questions came at the end of the survey. The median duration to complete the longer version of the instrument was 14.41 min (from 13.67 min in the US to 18.07 min in Chile). The median duration to complete the shorter version of the instrument was 9.15 min (from 7.45 min in Canada to 16.55 min in Pakistan).

### Participants

The final dataset consisted of 4958 (46.2% women) responses from 27 countries, ranging from 62 responses in Peru to 380 responses in the U.S. Gender participation was hugely varied, with women making up as few as 21.3% of participants in France, to 82.2% in Bosnia and Herzegovina. The median age of the entire sample was 38 (median of 34 in two countries to 46 in two countries). Of all participants, 78.8% had completed higher education. Most participants (71.4%) were employed full-time. Across countries, 30.1% of participants came from below-average or poor households, ranging from 17.5% in Pakistan to 51.6% in Peru. We then excluded participants with entries that did not align with our pre-registration requirements and tracked these changes in our “Exclusion Table” (Supplementary Materials, Table S3) which displays total participants removed and percent rate of change in each country. Comprehensive details on data inclusion are provided in the Supplementary Materials (Table S7a,b).

### Classification

The classification employed in the paper—positive deviants, low-income participants, and above average participants—is based on survey questions eliciting (1) participants’ financial situation in the household they grew up in, (2) their current income, (3) national income data from participant country of residence, and (4) the sample spread of income data from participant country of residence. Positive deviants are defined as adults who reported growing up in low-income households but who demonstrate a reasonable level of financial wellness in adulthood.

Specifically, to define the cut-off point, we calculated the midpoint between the average national income in each country and the median income within our country samples. This was done so that the cut off did not rely solely on nationally reported averages from each country, as these come from different sources and may not account for recent economic changes such as high rates of inflation.

As a result, our midpoint line sits above the national average and below our sample median. Positive deviants are thus defined as adults who reported growing up in low-income households and whose income falls above this line. Low-income individuals are also adults who reported growing up in low-income households but whose income falls below this line, which means that they started off in a low-income environment but were not able to achieve significantly higher incomes as adults. Everyone else was classified as above average and excluded from the analyses unless otherwise specified.

### Deviations from pre-registered plan

Due to the complexity of the study, primarily based on including countries with entirely different economic systems and standards, recent extreme inflation and related taxation policies, as well as differing availability of reliable income estimates, several critical adjustments had to be made, though did not appear to impact outcomes. We removed the income buffer zone (40th to 50th percentile) for individuals that were born low-income, as this was determined to only be appropriate in a small number of countries and would have resulted in excluding many entirely legitimate participants from analyses. We also excluded mental accounting as, after starting data collection, it was agreed that our measures simply did not test for nor elicit this bias. For posterity and any future attempts to utilize these data, we provide a comprehensive and annotated source and decision table for all countries, which will be posted with all pre-registration material, code, and data.

## Supplementary Information

Supplementary Information.

## Data Availability

All data will be posted open access via https://psyarxiv.com/mrxy6/ and in interactive form via https://public.tableau.com/app/profile/kai.ruggeri. We will post these only once an accepted version of all analyses is possible to avoid confusion based on version control.
