# Supplementary material for: The persistence of cognitive biases in financial decisions across economic groups
Source: Sci Rep. 2023 Jun 26;13:10329. doi: 10.1038/s41598-023-36339-2 (PMC10293260; doi:10.1038/s41598-023-36339-2)
Supplement: Supplementary file 1 — Supplementary Information. [file 41598_2023_36339_MOESM1_ESM.pdf]

SUPPLEMENTARY INFORMATION

The persistence of cognitive biases in financial decisions across economic groups

Kai Ruggeri*, Sarah Ashcroft-Jones*, Giampaolo Abate Romero Landini, Narjes Al-Zahli, Natalia Alexander, Mathias Houe Andersen, Katherine Bibilouri, Katharina Busch, Valentina Cafarelli, Jennifer Chen, Barbora Doubravová, Tatianna Dugué, Aleena Asfa Durrani, Nicholas Dutra, Eduardo Garcia-Garzon, Christian Gomes, Aleksandra Gracheva, Neža Grilc, Deniz Mısra Gürol, Zoe Heidenry, Clara Hu, Rachel Krasner, Romy Levin, Justine Li, Ashleigh Marie Elizabeth Messenger, Melika Miralem, Fredrik Nilsson, Julia Marie Oberschulte, Takashi Obi, Anastasia Pan, Sun Young Park, Daria Stefania Pascu, Sofia Pelica, Maksymilian Pyrkowski, Katherinne Rabanal, Pika Ranc, Žiga Mekiš Recek, Alexandra Symeonidou, Olivia Symone Tutuska, Milica Vdovic, Qihang Yuan, Friederike Stock

*Contributed equally & sharing first-authorship

Supplementary Methods

The data used for the analysis in this report is from the survey we created for our current study (mentioned in Main Manuscript). We used R 4.2.1 with RStudio 2022.07.1. List of packages we used: brms v. 2.17.0; ggplot2 v. 3.3.6; tidyverse v. 1.3.2; arm v. 1.12-2; visdat v. 0.5.3; janitor v. 2.1.0; corrplot v. 0.92 ; Hmisc v. 4.7-0; PerformanceAnalytics v. 2.0.4; ggstatsplot v. 0.9.4; scales v. 1.2.0; RColorBrewer v. 1.1-3; tidytext v. 0.3.3; Rmisc v. 1.5.1; ggnewscale v. 0.4.7; gplots 3.1.3; readr v. 2.1.2; wpa v. 1.8.0; sf v. 1.0-8; rnaturalearth v. 0.1.0; patchwork v. 1.1.2; freqtables v. 0.1.1; countrycode v. 1.4.0; glue v. 1.6.2; ggridge v. 0.5.3; tidybayes v. 3.0.2; Rcpp v. 1.0.9; ggflags v. 0.0.2; ggpubr v. 0.4.0, jmv v. 2.3.4; gridExtra v. 2.3; xts v. 0.12.1; zoo v. 1.8-10; Formula v. 1.2-4; survival v. 3.3-1; lattice v. 0.20-45; lme4 v. 1.1-29; Matrix v. 1.4-1; MASS v. 7.3-57; forcats v. 0.5.1; stringr v. 1.4.0; purrr v. 0.3.4; tidyr v. 1.2.0; tibble v. 3.1.8; cmdstanr v. 0.5.2 . All accessed on August 18th of 2022.

We created a table to track the changes made on the total number of participants (Table S3). We started the study with 5898 participants. We then removed participants for completion times that were deemed too quick, entries that did not align with pre-registration requirements, unreasonable high-income in relation to the country's median income, and based in implausible age. This resulted in a total of 4958 participants. With these values, we tracked the number of participants we lost as we excluded them from the data pool. Additionally, we calculated the percent rate of change for how much we lost per exclusion category. The total percent lost, percent lost from the initial start and exclusion category, of participants per country was also tracked. Systematic causes were not the primary reason for exclusion.

We computed the frequency ratios in relation to gender, age, higher education, full-time employment, annual income, and childhood household financial situation in both total dataset and country levels to mark for potential variances. The "Other" category in "Gender" includes answers from participants who preferred to not answer the question or identified as a gender other than "Man" or "Woman", and made up 2.99% of the total data set. The "Gender_Dif" column calculated the difference between male and female percentage within a country, with a value closer to 0% representing a more equal gender distribution in the sample. Age range was calculated by finding the difference between the oldest participant age and youngest participant age. We defined higher education as completion of a bachelor's degree or above. Our definition for full-time employment was working at least 30 hours per week. We mainly focused on the participants with full-time employment (all items used for this variable are presented in Table S2) for analysis since the age limitation for our survey was 30 years old, and we expect them to have a regular income. Participants considered born into low-income households self-identified their childhood financial household situation as either "poor" or "below average but not poor."

Supplementary Table S5 portrays both frequency of biases across countries and the total dataset of the biases. In total, we measured 10 cognitive biases. In order to determine presence of bias for three of the ten biases two conditions must be met. This was true for disposition effect (decrease/increase), framing effect (gain/loss), and loss aversion (gain/loss). The lowest and highest frequency of each bias to find potential variabilities between countries. Our findings indicate high variability in disposition bias as seen with Chile (23.4%) and Italy (60.9%), signifying Italy's high ability to make optimal decisions regardless of ambiguity. Overplacement and overestimation bias frequency excluded Bosnia and Herzegovina, Chile, Germany, Italy, Sweden, Turkey, and the United States due to differences in survey length.

Similarly, a more prominent difference is echoed in loss aversion bias scores between Greece (the lowest scoring country with 6.22%) and Serbia (the following lowest scoring country 33.6%), compared to Japan (67.3%) with the highest score. A similar trend appears in temporal discounting bias between Germany (11.0%) and Turkey (67.0%). As opposed to lower frequency scores of Greece in loss aversion bias and Germany in temporal discounting bias, Czech Republic (93.6%) in disposition decrease, Japan (91.7%) in frame gain, and Japan (90.4%) in loss aversion gain bias had adversely high frequency scores (those three measures mentioned were a part of calculating their respective biases). However, less variability appeared more frequently in the majority of the biases (e.g.; baseratefall, overestimation, loss aversion loss).

The analysis of the data for all countries shows that higher scores in disposition decrease (85.3%), frame gain (74.7%), and loss aversion gain (79.6%) imply substantial bias, indicating that such correlation exists across countries. Total scores for all of the countries in frame gain (74.7%) and frame loss (67.8%) frequencies were relatively divided across individual countries, suggesting that individuals favor an opportunity framed as a gain as compared to a loss. Similar

patterns emerge in loss aversion gain (79.8%) and loss aversion loss (68.7%) measures as well. In contrast, scores taken from the total dataset, consisting of data from all countries in disposition decrease (85.0%) and increase (52.0%) demonstrate greater utility with the belief that its value will increase—despite it being unlikely to—with dissimilar expectations towards high probability stock. After analyzing our collected data, we excluded South Africa and Israel due to the two not meeting our minimum criteria of participants. Bosnia and Herzegovina, Chile, Germany, Italy, Sweden, Turkey, and the United States were excluded from overestimation and overplacement counts and percentages.

The chi-squared tests were conducted for the data gathered in all countries for all biases. Comparing positive deviants and below-average participants did not show any significant differences for any of the tested biases (results available in Table S8).

We calculated the difference between the overplacement score and the number of biases and compared the mean difference of 3 groups; low-income, positive deviants, and above-average. Independent-measures ANOVA shows no significant difference between these 3 groups ($F(2)=0.281$, $P=0.755$). We excluded 7 countries (Bosnia, Chile, Germany, Italy, Sweden, Turkey, USA) from the calculation since they had 10 biases rather than 5. We also attempted 20 separate one-way ANOVAs for independent measures for each country and did not find significant differences between those groups in any country.

When calculating the binomial logistic regression models to analyze whether the country of residence or being a positive deviant can predict the presence of each cognitive bias, we excluded the same countries as we did in the ANOVA, because of differences in the number of survey items that lead to different scales for the overestimation and overplacement items.

We conducted ten binomial logistic regressions to predict the presence of cognitive bias based on income group and country of residence. We calculated Tjur's $R2$ as a measure of variance explained by the model. The model with the dependent variable disposition explained 5 %, ambiguity 5%, base rate effect 7%, extremeness 4%, temporal discounting 8%, framing 4 % and loss aversion 8 %. Because the predictors in the analysis were categorical, we set the low-income group as the reference group for the predictor income group and the USA as the reference group for the predictor country. Prediction coefficients for the income group were not significant in any of ten logistic regressions; positive deviants were equally likely to exhibit cognitive bias compared to the low-income individuals. Table 1 (Main Text) provides coefficients.

As a robustness check, we then ran Bayesian logistic regressions for each of the 10 biases to complement our original binomial logistic regressions. As expected, we notice that the credible intervals (computed at the 95% level) generated through the Bayesian analyses are consistent with the confidence intervals (also originally computed at the 95% level) for both the intercept

and all the variables included in the models (i.e., positive deviance indication and country of residence). We conclude that this additional analysis provides further evidence that rates of cognitive biases do not seem to differ between positive deviants and low-income adults. See Table 6 for credible intervals from all Bayesian logistic regressions.

Figure S1. Mean differences between the overplacement score and the number of biases for low income, positive deviance and above average group

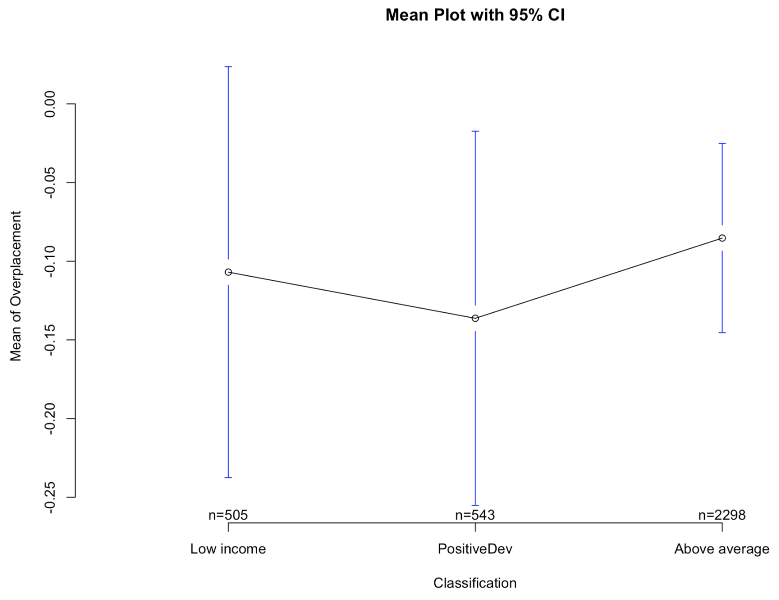

Data checks

We reviewed all entries to flag any responses (regarding employment, income, savings, debt, and text entries for race and gender) that would have been excluded or included for mistaken reasons by our code (i.e., an exclusion for 90% of income cut-off). Participants were asked to provide their income (income1), debt (income2), and savings/assets (income3). Participants were also given the option to write in their gender, race, and ethnicity on the survey. We reviewed and confirmed that the responses were kept for appropriate responses regarding each matter, especially income. Although the flagging process was not used for reviewing the dataset for this project, we believe that this can be used later and as recommendations for future projects for those who would want to use the same dataset. Refer to File "Data Checking Spreadsheet" for further information regarding participant ID and inclusion/exclusion explanation. All flagged data in our file are completed survey entries.

Surveys

Two versions of our Qualtrics survey were distributed: 7 countries (Bosnia, Chile, Germany, Italy, Sweden, Turkey, U.S.) received 30 decision-making questions, while twenty countries received 15 of the 30 original questions. Our data and analyses were only based on the same 15 questions received by all countries.

Supplementary Tables

Table S1. Cognitive biases of interest with brief definition

**Ambiguity Effect**	The tendency to avoid options that are ambiguous, preferring less ambiguous alternatives. Certainty is prioritized, even if a more ambiguous alternative has equal–or better–expected returns.
**Base Rate Fallacy**	Placing greater value on contingent information or secondary probabilities than on the full information.
**Category Size Bias**	A preference for choices that come from larger, more likely categories, even if certainty and risk are the same in smaller categories.
**Disposition Effect**	A financial phenomenon in which investors tend to hold losing assets for too long and sell winning assets too early.
**Framing Effect**	Differential preferences are elicited based on changing how the same information is presented in different ways.
**Loss Aversion**	Being more sensitive to losses compared to gains, resulting in a preference to avoid losses over acquiring equivalent gains.
**Overconfidence Bias**	Tendency of overestimating the accuracy of our own knowledge and skills. This includes two subcategories, Overestimation and Overplacement. The second one is in relation to others.
**Temporal Discounting**	Choosing smaller, immediate financial gains over larger, delayed gains.
**Extremeness aversion**	A tendency to avoid extreme options in choice scenarios.

Table S2. Additional variables used in the study

**Age**	Variable ranging from 30 to 100
**Education Completed**	Variable presenting the highest level of education attained. This variable presented five (or more depending on country?) groups: "Primary education or less," "Secondary education," "Technical or Vocational education," "Bachelor studies or equivalent," and "Higher or Graduated degree."
**Employment**	Variable representing current employment status. Presented the following levels: "Employed full-time" (which included military service), "Employed part-time," "Self-employed," "Not in paid employment but looking," "Not in paid employment for personal reasons," "Full-time student," and "Retired."
**Gender**	Variables presenting four levels: "Male," "Female," "I prefer not to answer," and "Other." "Other" represented all individuals not categorizing themselves into strict male to female categories.
**Individual income**	Variable representing self-reported monthly or annual income from all sources before taxes.
**Individual debt**	Variable representing self-reported total debt balance at the end of 2021 (including credit cards, student loans, and other credit not including housing or monthly bills, except if overdue).
**Individual assets**	Variable representing self-reported total assets including savings, retirement plans, investments accounts, and home equity at the end of 2021.

Table S3: Exclusion data

**Residence**	Initial numer of participants	Number of participants after speed-based exclusion	Percentage of participants removed based on speed	Number of participants after removal based on unreasonably low income	Percentage of participants removed based on unreasonably low income	Number of participants after removal based on alignment with pre-registration requirements	Percentage of participants removed based on alignment with pre-registration requirements	Number of participants after removal based on unreasonably high income in relation to the median	Percentage of participants removed based on unreasonably high income in relation to the median	Participants after removal based on implausibly high age	Percentage of participants removed based on implausibly high age	Total number of participants lost	Total percentage of participants lost
**Bosnia and Herzegovina**	400	400	0.0	343	14.2	343	0.00	342	0.29	342	0.0	58	14.5
**Brazil**	238	238	0.0	215	9.7	213	0.93	207	2.82	207	0.0	31	13.0
**Canada**	264	264	0.0	221	16.3	221	0.00	221	0.00	221	0.0	43	16.3
**Chile**	80	80	0.0	64	20.0	64	0.00	64	0.00	64	0.0	16	20.0
**Czech Republic**	247	246	0.4	217	11.8	217	0.00	216	0.46	216	0.0	31	12.6
**Denmark**	233	233	0.0	200	14.2	198	1.00	198	0.00	198	0.0	35	15.0
**France**	118	116	1.7	93	19.8	91	2.15	91	0.00	89	2.2	29	24.6
**Germany**	203	201	1.0	183	9.0	183	0.00	182	0.55	182	0.0	21	10.3
**Greece**	272	272	0.0	197	27.6	193	2.03	193	0.00	193	0.0	79	29.0
**Ireland**	109	108	0.9	95	12.0	95	0.00	95	0.00	95	0.0	14	12.8
**Italy**	222	220	0.9	204	7.3	203	0.49	203	0.00	202	0.5	20	9.0
**Japan**	305	301	1.3	251	16.6	251	0.00	251	0.00	251	0.0	54	17.7
**North Macedonia**	154	150	2.6	124	17.3	123	0.81	123	0.00	123	0.0	31	20.1
**Oman**	155	155	0.0	137	11.6	137	0.00	137	0.00	137	0.0	18	11.6
**Pakistan**	101	101	0.0	66	34.7	65	1.52	63	3.08	63	0.0	38	37.6
**Peru**	82	81	1.2	64	21.0	62	3.12	62	0.00	62	0.0	20	24.4
**Poland**	205	204	0.5	158	22.5	156	1.27	154	1.28	154	0.0	51	24.9
**Portugal**	352	351	0.3	300	14.5	300	0.00	299	0.33	299	0.0	53	15.1
**Romania**	113	112	0.9	83	25.9	82	1.20	81	1.22	81	0.0	32	28.3
**Serbia**	215	215	0.0	154	28.4	152	1.30	152	0.00	152	0.0	63	29.3
**Slovenia**	330	330	0.0	286	13.3	285	0.35	283	0.70	283	0.0	47	14.2
**South Korea**	156	149	4.5	132	11.4	132	0.00	132	0.00	132	0.0	24	15.4
**Sweden**	262	261	0.4	246	5.8	246	0.00	245	0.41	242	1.2	20	7.6
**Taiwan**	216	210	2.8	169	19.5	169	0.00	169	0.00	169	0.0	47	21.8
**Turkey**	212	211	0.5	200	5.2	200	0.00	200	0.00	200	0.0	12	5.7
**United Kingdom**	261	256	1.9	221	13.7	221	0.00	221	0.00	221	0.0	40	15.3
**United States**	393	393	0.0	382	2.8	382	0.00	380	0.52	380	0.0	13	3.3
**Total**	5898	5858	1.6	5005	30.4	4984	1.15	4964	0.83	4958	0.3	940	15.9

Table S4. Bayesian Meta-Analysis model results

Model	Group-Level Effects: τ = sd(Intercept) Estimate	Population-Level Effects: SMD = Intercept Estimate	Sample Size
18 countries: biases [0-10] - full sample	0.22	4.87	3194
18 countries: biases [0-10] - pdev only	0.43	4.84	528
25 countries: biases [0-8] - full sample	0.23	3.98	4806
25 countries: biases [0-8] - pdev only	0.40	4.95	765

Table S5. ANOVA comparing mean differences in bias results in three groups (low-income, above average and positive deviants) in each country

Country (df)	Pr(>F)	F Value
Brazil (2, 204)	0.589	0.531
Canada (2, 218)	0.395	0.933
Czech Republic (2, 213)	0.491	0.713
Denmark (2, 195)	0.487	0.722
France (2, 86)	0.892	0.114
Greece (2, 190)	0.901	0.104
Ireland (2, 92)	0.532	0.634
Japan (2, 248)	0.664	0.410
North Macedonia (2, 120)	0.174	1.776
Oman (2, 134)	0.553	0.595
Pakistan (2, 60)	0.830	0.187
Peru (2, 59)	0.096	2.435
Poland (2, 151)	0.310	1.181
Portugal (2, 296)	0.710	0.342
Romania (2, 78)	0.831	0.185
Serbia (2, 149)	0.369	1.002
Slovenia (2, 280)	0.179	1.734
South Korea (2, 129)	0.772	0.259
Taiwan (2, 166)	0.393	0.940
United Kingdom (2, 218)	0.916	0.087

Table S6: Robustness check: Bayesian logistic regressions credible intervals for predicting biases by residence and income group

	Disposition	Ambiguity	Baserate	Categorysize	Extremeness	Temporal discounting	Framing	Loss aversion	Overplacement	Overestimation
(Intercept)	[-0.832, -0.098]	[0.354, 1.129]	[-0.204, 0.568]	[-0.977, -0.225]	[-0.278, 0.454]	[-1.443, -0.58]	[-0.089, 0.643]	[0.233, 0.992]	[-1.043, -0.005]	[-0.591, 0.432]
Positive deviance	[-0.273, 0.165]	[-0.388, 0.076]	[-0.174, 0.27]	[-0.214, 0.216]	[-0.217, 0.223]	[-0.428, 0.078]	[-0.266, 0.159]	[-0.336, 0.108]	[-0.138, 0.407]	[-0.254, 0.277]
Bosnia and Herzegovina	[-1.335, -0.077]	[-0.564, 0.623]	[0.368, 1.635]	[-0.327, 0.8]	[-1.81, -0.617]	[-0.17, 1.069]	[-0.966, 0.147]	[-0.579, 0.601]	NA	NA
Brazil	[-0.579, 0.6]	[0.156, 1.55]	[0.143, 1.389]	[-0.239, 0.919]	[-0.489, 0.666]	[0.127, 1.327]	[-1.02, 0.11]	[-0.531, 0.698]	[0.251, 1.609]	[-1.149, 0.227]
Canada	[-0.172, 1.03]	[-0.715, 0.541]	[-0.867, 0.328]	[0.017, 1.216]	[0.077, 1.334]	[-1.1, 0.406]	[-0.454, 0.734]	[-1.354, -0.127]	NA	NA
Chile	[-2.928, -0.249]	[0.078, 2.87]	[0.332, 2.636]	[-0.964, 0.942]	[-1.201, 0.659]	[-0.755, 1.282]	[-1.823, 0.123]	[-1.715, 0.19]	NA	NA
Czech Republic	[-0.519, 0.662]	[-0.732, 0.497]	[0.665, 2.064]	[-0.256, 0.944]	[-0.075, 1.115]	[-0.603, 0.742]	[-1.196, -0.047]	[-0.119, 1.167]	[-0.677, 0.732]	[-1.704, -0.27]
Denmark	[-0.141, 1.189]	[-0.973, 0.379]	[-0.654, 0.643]	[-0.589, 0.739]	[-0.845, 0.419]	[-1.349, 0.3]	[-0.869, 0.455]	[-0.763, 0.572]	[-0.485, 1.013]	[-0.869, 0.615]
Germany	[-0.298, 1.073]	[-0.397, 1.17]	[-0.714, 0.649]	[-0.492, 0.963]	[-0.246, 1.215]	[-2.602, -0.262]	[-1.918, -0.414]	[-1.164, 0.244]	NA	NA
Greece	[-0.069, 1.289]	[-0.067, 1.483]	[0.708, 2.407]	[-1.042, 0.403]	[-0.953, 0.361]	[0.011, 1.423]	[-0.698, 0.597]	[-5.78, -2.666]	[0.425, 1.976]	[0.04, 1.534]
Ireland	[0.717, 2.46]	[-0.792, 0.784]	[-1.517, 0.08]	[-1.793, 0.06]	[-0.499, 1.063]	[-0.684, 1.047]	[-1.252, 0.274]	[-1.69, -0.123]	[-1.603, 0.299]	[-0.775, 0.897]
Italy	[0.531, 1.967]	[-0.444, 1.046]	[-0.275, 1.121]	[-0.651, 0.798]	[-1.031, 0.364]	[-1.19, 0.471]	[-1.246, 0.102]	[-1.608, -0.202]	NA	NA
Japan	[-0.276, 0.846]	[-0.716, 0.434]	[-1.341, -0.177]	[-1.142, 0.054]	[-0.548, 0.558]	[-1.868, -0.318]	[-0.265, 0.856]	[-0.212, 0.947]	[-1.185, 0.182]	[-0.312, 1.008]
North Macedonia	[-0.83, 0.633]	[-0.495, 1.048]	[0.185, 1.804]	[-0.867, 0.638]	[-0.451, 1]	[-0.14, 1.361]	[-1.107, 0.274]	[-1.322, 0.101]	[-0.482, 1.097]	[-0.877, 0.708]
Oman	[-0.476, 0.915]	[0.006, 1.694]	[0.319, 1.983]	[-0.271, 1.085]	[-0.679, 0.647]	[0.02, 1.452]	[-0.94, 0.446]	[-1.236, 0.145]	[-1.193, 0.421]	[-1.272, 0.316]
Peru	[-0.975, 0.641]	[0.186, 2.264]	[0.371, 2.364]	[0.586, 2.245]	[-0.895, 0.692]	[0.46, 2.076]	[-1.416, 0.142]	[-1.17, 0.385]	[-0.173, 1.577]	[-0.792, 0.907]
Poland	[-0.427, 1.067]	[-0.835, 0.761]	[-0.802, 0.764]	[-0.957, 0.685]	[-0.272, 1.269]	[-0.343, 1.358]	[-1.319, 0.212]	[-1.642, -0.057]	[-0.758, 1.003]	[-1.156, 0.606]
Portugal	[-0.211, 0.828]	[0.388, 1.74]	[0.045, 1.184]	[-0.95, 0.184]	[0.043, 1.134]	[-1.071, 0.26]	[-0.833, 0.204]	[-0.912, 0.186]	[0.016, 1.298]	[-0.37, 0.918]
Romania	[-1.109, 0.723]	[-0.55, 1.385]	[-0.756, 1.003]	[0.221, 2.029]	[-0.943, 0.764]	[-0.161, 1.648]	[-0.438, 1.334]	[-0.766, 1.014]	[-0.453, 1.353]	[-1.387, 0.464]
Serbia	[-1.463, 0.306]	[-1.497, 0.073]	[0.261, 2.137]	[-0.832, 0.804]	[-0.27, 1.284]	[-0.158, 1.468]	[-1.746, -0.169]	[-2.109, -0.46]	[-0.459, 1.217]	[-0.313, 1.39]
Slovenia	[-0.714, 0.432]	[-0.638, 0.543]	[-0.103, 1.068]	[0.214, 1.31]	[-0.042, 1.111]	[-0.516, 0.733]	[-0.206, 0.925]	[-0.364, 0.79]	[-0.833, 0.511]	[-1.888, -0.434]
South Korea	[-0.599, 0.8]	[0.503, 2.575]	[-0.43, 1.006]	[0.712, 2.19]	[-0.299, 1.13]	[-0.502, 1.048]	[-0.244, 1.198]	[-0.733, 0.7]	[-1.107, 0.525]	[-1.354, 0.274]
Sweden	[0.149, 1.295]	[-0.976, 0.142]	[-0.254, 0.894]	[-0.298, 0.827]	[-0.314, 0.828]	[-1.339, 0.124]	[-1.076, 0.04]	[-0.452, 0.688]	NA	NA
Taiwan	[-0.709, 0.717]	[-0.351, 1.309]	[-0.665, 0.802]	[-0.905, 0.622]	[-0.47, 1.03]	[-2.408, -0.159]	[-1.237, 0.202]	[-1.768, -0.318]	[-3.138, -0.704]	[-0.467, 1.155]
Turkey	[0.213, 1.722]	[-0.155, 1.575]	[-0.933, 0.547]	[-0.134, 1.357]	[-1.465, 0.061]	[0.907, 2.557]	[-1.93, -0.304]	[-1.299, 0.219]	NA	NA
United Kingdom	[0.003, 1.018]	[-0.852, 0.198]	[-0.65, 0.408]	[-1.086, 0.053]	[-0.266, 0.761]	[-1.215, 0.105]	[-0.2, 0.84]	[-0.476, 0.598]	[-0.565, 0.702]	[-1.219, 0.05]

Note on income group: participants in analysis are either low-income or positive deviants. The 'Positive Deviance' variable in the table captures the behavior of positive deviants, with low-income as the baseline(high-income participants are not included in this analysis). Note on residence: all country variables reflect participants' country of residence, with the USA as the baseline (for disposition to loss aversion; Canada as the baseline for Overplacement and Overestimation since the USA is excluded from those analyses along with Bosnia & Herzegovina, Chile, Germany, Italy, Sweden, and Turkey.

Table S7a. Number of Observations, Total and per Income Group

	Total	Above Average*	Total excl. Above Average*	Low Income	Positive Deviants
Bosnia and Herzegovina	342	262	80	45	35
Brazil	207	132	75	43	32
Canada	221	155	66	29	37
Chile	64	42	22	11	11
Czech Republic	216	143	73	36	37
Denmark	198	147	51	26	25
Germany	182	138	44	25	19
Greece	193	142	51	26	25
Ireland	95	61	34	14	20
Italy	202	157	45	21	24
Japan	251	163	88	68	20
North Macedonia	123	82	41	23	18
Oman	137	91	46	22	24
Peru	62	30	32	10	22
Poland	154	121	33	13	20
Portugal	299	204	95	47	48
Romania	81	55	26	13	13
Serbia	152	119	33	13	20
Slovenia	283	197	86	25	61
South Korea	132	90	42	12	30
Sweden	242	161	81	35	46
Taiwan	169	130	39	20	19
Turkey	200	164	36	14	22
United Kingdom	221	114	107	50	57
United States	380	248	132	52	80
Total	4806	3348	1458	693	765

* Participants with income above average are excluded from the analyses unless otherwise specified, which is why here we also report the total number of observations excluding them (i.e., only including low-income participants and positive deviants).

Table S7b. Demographics by country (total in bottom row)

Residence	Number of participants	Number of males	Percentage of male participants	Female Count	Percentage of female participants	Other Count	Percentage of participants of other genders	Gender Dif (negative values indicate more women)	Age Median	Age Range	Number of participants who have higher education	Percentage of participants who have higher education
Bosnia and Herzegovina	342	52	15.2	281	82.2	9	2.63	-67	38	37	253	74
Brazil	207	143	69.1	59	28.5	5	2.42	40.6	35	51	167	80.7
Canada	221	126	57	89	40.3	6	2.71	16.7	37	54	155	70.1
Chile	64	32	50	31	48.4	1	1.56	1.56	36	34	52	81.2
Czech Republic	216	109	50.5	97	44.9	10	4.63	5.56	37	46	144	66.7
Denmark	198	116	58.6	79	39.9	3	1.52	18.7	42	55	177	89.4
France	89	69	77.5	19	21.3	1	1.12	56.2	34	38	84	94.4
Germany	182	90	49.5	83	45.6	9	4.95	3.85	40	49	123	67.6
Greece	193	105	54.4	83	43	5	2.59	11.4	44	43	175	90.7
Ireland	95	55	57.9	39	41.1	1	1.05	16.8	37	43	89	93.7
Italy	202	98	48.5	93	46	11	5.45	2.48	40	47	141	69.8
Japan	251	113	45	133	53	5	1.99	-7.97	42	46	143	57
North Macedonia	123	60	48.8	61	49.6	2	1.63	-0.813	40	39	108	87.8
Oman	137	61	44.5	63	46	13	9.49	-1.46	34	55	115	83.9
Pakistan	63	34	54	29	46	0	NA	7.94	37	34	61	96.8
Peru	62	36	58.1	23	37.1	3	4.84	21	46	39	50	80.6
Poland	154	77	50	68	44.2	9	5.84	5.84	35	47	153	99.4
Portugal	299	144	48.2	149	49.8	6	2.01	-1.67	36	36	250	83.6
Romania	81	46	56.8	33	40.7	2	2.47	16	45	42	58	71.6
Serbia	152	83	54.6	66	43.4	3	1.97	11.2	41	51	108	71.1
Slovenia	283	171	60.4	109	38.5	3	1.06	21.9	37	42	204	72.1
South Korea	132	80	60.6	51	38.6	1	0.758	22	45	37	126	95.5
Sweden	242	164	67.8	74	30.6	4	1.65	37.2	36	60	157	64.9
Taiwan	169	73	43.2	88	52.1	8	4.73	-8.88	44	39	151	89.3
Turkey	200	97	48.5	96	48	7	3.5	0.5	46	47	191	95.5
United Kingdom	221	125	56.6	90	40.7	6	2.71	15.8	35	34	153	69.2
United States	380	161	42.4	204	53.7	15	3.95	-11.3	40.5	59	321	84.5

Table S8. Reported values of the chi-square tests for aggregated data per bias.

n_{obs}=1030 (366 low-income, 664 positive deviants)

Type of bias	χ^2 (*df*=1)	*P* value
Ambiguity bias	2.09	0.15
Base rate fallacy bias	<0.01	0.96
Category size bias	0.01	0.91
Disposition bias	0.04	0.83
Extremeness aversion	0.61	0.43
Framing	0.05	0.82
Loss aversion	0.11	0.74
Overestimation	1.75	0.19
Overplacement	0.03	0.87
Temporal discounting bias	<0.01	1.00

Table S9: Survey Item Questions

Bias	Item
Loss aversion	Gain: Which option do you prefer? a) an 80% chance of gaining $4,000 (20% chance of gaining 0), b) a 100% guarantee of gaining $3,000<br><br>Loss: Which option do you prefer? a) an 80% chance of losing $4,000 (20% chance of losing 0), b) a 100% guarantee of losing $3,000
Base Rate Fallacy	Which company would you prefer to invest in?<br>A. Company 1 is in an industry where only 10% of companies are successful. The leaders work extremely hard, employees are highly skilled and committed, and they do not take risks or waste any time/resources.<br>B. Company 2 is in an industry where 90% of companies are successful. The leadership is about average, employees have only basic skills, and there are no special restrictions on how time or resources are used.
Category Size Bias	If you entered a drawing to win $1,000, which option would you prefer?<br>A. 10 winning tickets out of 100<br>B. 1 winning ticket out of 10
Overconfidence (overestimation)	Throughout this survey, you have been presented XX items, of which YY had measurably greater or worse options. Out of the YY questions that had a better or worse option, how many times do you think you chose the better option?
Overconfidence (overplacement)	Throughout this survey, you have been presented XX items, of which YY had measurably greater or worse options. Out of the YY questions that had a better or worse option, how many times do you think the average person chose the better option?
Disposition effect	1: You have chosen to invest $10,000 in a company. After three months, the stock value has doubled and is now worth $20,000. You can either sell now or leave them, but you cannot withdraw again for 3 months. Which would you prefer?<br>A. Keep<br>B. Sell<br>2: You have chosen to invest $10,000 in a company. After three months, the stock price has cut in half. Your stocks are therefore now worth $5,000. You can either sell now or leave them, but you cannot withdraw again for 3 months. Which would you prefer?<br>A. Keep<br>B. Sell
Temporal discounting	Which one would you prefer?<br>C. Receive $5,000 now<br>D. Receive $6,000 in 12 months<br>E. Receive $500 every month for 12 months
Mental accounting	Which one would you prefer?<br><br>A. A 1000 euro apartment that currently rents for 1000<br><br>B. A 1200 euro apartment that currently rents for 1000
Framing effect	Gain: If you had $1,000 to save or invest, which would you prefer?<br>A. A 50% chance to gain an additional $1,000 (50% chance of gaining 0 beyond what you already have)<br>B. A 100% guarantee of gaining an additional $500<br>Lose: If you had $2,000 to save or invest, which would you prefer?<br>A. A 50% chance you will lose $1,000 (50% chance of losing 0)<br>B. A 100% chance you will lose $500

Supplementary Data

All supplementary data is available under the preregistration link: osf.io/wj9yn
